# 621 Efforts Used to Increase Nursing Barcode Scan Rates on a Burn Unit

**DOI:** 10.1093/jbcr/iraf019.250

**Published:** 2025-04-01

**Authors:** Hannah Thielges, Bradley Rogers, Ellie Thibodeau

**Affiliations:** Regions Hospital Burn Center; Regions Hospital Burn Center; Regions Hospital Burn Center

## Abstract

**Introduction:**

Medication administration errors pose significant risks to patient safety, particularly in high-acuity settings such as burn units. Barcode medication administration (BCMA) systems are designed to reduce such errors. According to DeYoung (2009), medication-related errors decreased by 56% in a medical intensive care unit once a BCMA system was utilized robustly. Unfortunately, nursing compliance with scanning protocols can be inconsistent. Increasing barcode scan rates is critical to ensuring patient safety and improving outcomes. In this project, the goal is for each nurse to achieve a scan rate of both the patient wristband and the medication label 95% of the time or more. Moving forward, “met goal” in this project refers to nursing staff achieving this percentage. This project aims to evaluate the effectiveness of targeted interventions designed to increase the barcode scan rate to meet the stated goal by comparing scan rates before and after the implementation of the interventions. This unit was identified to have low BCMA compliance in the months leading up to this project (January, February, and March 2024) with only 58%, 33%, and 50%, respectively, of nursing staff meeting goal.

**Methods:**

In this quality improvement project, a mixed-methods approach was employed throughout an eight month period (April through December of 2024) on a burn unit. Interventions included staff education on BCMA importance via huddles, posting individuals’ BCMA rates publicly, and the revision of workflows to address barriers, such as the implementation of smartphones with access to the electronic health records and scanning capabilities. Pre- and post-intervention data on scan rates were collected through the electronic health record.

**Results:**

The final analysis will include results from April 2024 to December 2024. Efforts to increase scanning compliance were implemented starting in April 2024. The percentage of staff that met goal in April and May are 52% and 48% respectively. The existing data from June, July, August, and September 2024 shows a modest improvement in staff meeting goal to 67%, 68%, 67%, and 83% respectively.

**Conclusions:**

This project could demonstrate the efficacy of education via huddles, posting medication scan rates, and the implementation of smartphones as tools to increase nurse compliance with barcode scanning on a burn unit. Continuous BCMA monitoring and support for nurses, along with addressing technological limitations, are essential to sustaining high compliance and ensuring optimal patient safety.

**Applicability of Research to Practice:**

In the current healthcare system where safety is the top priority, these initiatives could assist nurses in increasing BCMA compliance and preventing errors.

**Funding for the Study:**

N/A

